# Mn–Sb Co-doped SnO_2_ nanoparticles as efficient photocatalysts for tetracycline degradation

**DOI:** 10.1039/d6ra01125j

**Published:** 2026-04-02

**Authors:** Roomul Mushtaq, Towseef Ahmad, Farhana Wani, Kamila Kamili, Showket Ahmad Bhat, Adil Nazeer, Musheer-ul haq, Mohd Zubair Ansari

**Affiliations:** a Department of Physics, National Institute of Technology Srinagar Hazratbal Srinagar Jammu and Kashmir 190006 India showketbht7@gmail.com mohdzubair@nitsri.ac.in; b CRFC, National Institute of Technology Srinagar Hazratbal Srinagar Jammu and Kashmir 190006 India

## Abstract

The increasing presence of antibiotic contaminants such as tetracycline in aquatic environments poses a serious ecological and public health challenge, demanding efficient remediation strategies. In this work, Mn–Sb co-doped SnO_2_ nanoparticles were synthesized using a cost-effective sol–gel method to enhance the photocatalytic performance of SnO_2_. The Sb concentration was fixed at 4%, while Mn doping was varied from 0–8% to investigate its influence on structural, optical, and catalytic properties. Comprehensive characterization techniques including X-ray diffraction (XRD), Raman spectroscopy, FTIR, FESEM-EDS, UV-visible spectroscopy, X-ray photoelectron spectroscopy (XPS), and BET surface area analysis confirmed the successful incorporation of dopants into the SnO_2_ lattice. Structural analysis revealed the formation of a tetragonal rutile phase with a reduction in crystallite size from 36.55 to 24.54 nm upon Mn doping. Optical studies showed band-gap modulation from 3.37 to 3.24 eV at moderate Mn concentrations, while higher doping levels caused band-gap widening due to the Burstein–Moss effect. BET analysis demonstrated an increase in surface area from 70.84 to 82.49 m^2^ g^−1^, while XPS results indicated an increased concentration of oxygen-vacancy defects that facilitate charge separation. Photocatalytic experiments for tetracycline degradation under Xe-lamp irradiation revealed that the optimally doped MATO4% catalyst achieved the highest degradation efficiency of 79.5% with a rate constant of 0.0125 min^−1^. The enhanced photocatalytic performance is attributed to defect-induced charge separation, improved surface area, and optimized Mn doping. These findings highlight Mn–Sb co-doped SnO_2_ as a promising photocatalyst for antibiotic pollutant remediation and provide insights into defect engineering strategies for improving photocatalytic materials.

## Abstract 2

The increasing presence of antibiotic contaminants such as tetracycline in aquatic environments poses a serious ecological and public health challenge, demanding efficient remediation strategies.

## Graphical abstract

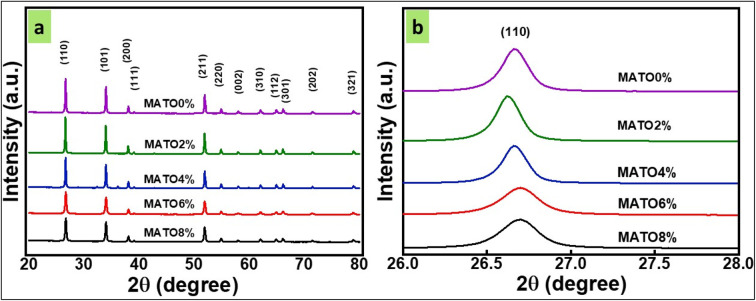

## Introduction

In recent decades, the pollution of aquatic and soil ecosystems with antibiotics, has emerged as a major public health concern due to its potential negative impacts on human health. Medical substances hold significant importance in modern society, spanning a diverse range of uses from healthcare to beauty products. There is a growing recognition of the presence of residues from personal care products and medicines in aquatic and terrestrial environments, resulting in their designation as a new type of contaminant.^1^ This matter has garnered considerable public interest and sparked extensive research and investigation. Among the various antibiotics, tetracycline, a well-known antibiotic, has been consistently detected and quantified in a range of typical regional environments, including landfills, mariculture sites, drinking water sources, and groundwater. It infiltrates the ecosystem *via* the excretion of unmetabolized materials from animals, the improper disposal of unused medications, and contamination from wastewater. Owing to its considerable solubility, it has the potential to leach into aquatic environments and soil, thereby enhancing its long-term persistence.^2^ Extended exposure to residual tetracycline at low concentrations may promote the development of antibiotic-resistant bacteria, contribute to the development of drug resistance, and facilitate the emergence of antibiotic resistance genes in microorganisms.^3^ The residual tetracycline has the potential to affect both the growth and metabolic processes of microorganisms, as well as alter the composition of the microbial community in the ecosystem.^4^ Tetracycline has a significant impact on the composition and functionality of microbial communities within soil ecosystems, which are crucial for nutrient cycling. Elevated levels of tetracycline were observed to diminish soil enzyme activity, obstruct nitrogen fixation, and facilitate the emergence of antibiotic-resistant bacteria. Research findings indicated that tetracycline residues extended their half-life in soils to more than 60 days. Furthermore, even post-degradation, specific metabolites continue to exhibit bioactivity, sustaining toxic effects and contributing to the selection pressures for microbial resistance.^3^ Therefore, developing effective and cost-efficient treatment methods to remove residual tetracycline from the environment is essential.

Various methods can be employed to degrade tetracycline, including biological, electrochemical, photocatalysis, and advanced oxidation processes. Using enzymatic processes such as hydroxylation, demethylation, and ring breakage, bacteria and fungi break down tetracycline into less toxic forms as part of the biological degradation process. Tetracycline is also rapidly broken down by the hydroxyl and sulphate radicals produced by the advanced oxidation process. Oxidative radicals generated by electrochemical techniques facilitate the removal of tetracycline.^5^ Photocatalysis is also an effective method for degrading tetracycline.

This study examines the application of doped semiconductor SnO_2_ nanoparticles as photocatalysts under Xe-lamp irradiation. Because SnO_2_ is a wide-bandgap semiconductor (∼3.6 eV), its photocatalytic activity is mainly activated by the ultraviolet (UV) component of the irradiation spectrum. In one of our previous studies, we found that 4% Sb-doped SnO_2_ is a better photocatalyst for the degradation of methylene blue dye.^6,7^ This study focuses on the co-doping of 4% Sb-doped SnO_2_ with Mn, examining its structural, morphological, optical, and compositional properties in relation to the photocatalytic degradation of tetracycline. SnO_2_ is a highly effective wide-band material (∼3.6 eV) characterised by its excellent chemical stability, low toxicity, and transparency, positioning it as a promising candidate for photocatalysis.^6,8,9^ The intrinsic characteristics, such as elevated mobility, substantial surface-to-volume ratio at the nanoscale, facilitate charge transfer processes and surface reactions. Nonetheless, in pure SnO_2_, the swift recombination of photo-generated electron–hole pairs constrains its photocatalytic capabilities.^10^ One method to address the issue is doping, which is expected to enhance light absorption and reduce electron–hole recombination. Consequently, following the confirmation of 4% Sb doping in SnO_2_ (ref. 6) Mn is co-doped and optimised to enhance the effectiveness of the SnO_2_ photocatalyst.^11^ The single-doped SnO_2_ has been verified for various characterisations and applications as per existing literature. For example, the electrochemical stability of the Sb–Mn co-doped electrode, as verified by Cairu S. *et al.*^12^ Mn-doped SnO_2_ was prepared by the chemical precipitation method to enhance the optical property of the bare SnO_2_.^13,14^ Congo red degradation was done using Mn-doped SnO_2_ prepared through the chemical method.^15,16^ Magnetic and optical properties of Mn-doped SnO_2_ were studied at different annealing temperatures.^17^ Uniformly spherical and monodisperse zinc-doped tin oxide nanoparticles were synthesised for optical and electronic applications.^8^

## Experimental section

### Synthesis

The synthesis of Sb-doped SnO_2_ and Mn–Sb-doped SnO_2_ nanostructures was done using a cost-effective sol–gel method. The dopant concentration of Sb for all the sample series was kept constant at 4%, while the Mn concentration was varied as 0%, 2%, 4%, 6%, and 8%. The synthesis was conducted using analytical-grade chemical reagents. In a 100 mL beaker, 40 mL of ethanol and 10 mL of deionised water were used as solvents to dissolve SnCl_4_·2H_2_O, SbCl_3_, and MnCl_2_·4H_2_O, forming the solutions. This solution was prepared at a temperature of 70 °C using a hot plate with a stirrer. During stirring, the ammonia was carefully added.

The precursor solution was prepared at 70 °C under continuous magnetic stirring on a hot plate. Aqueous ammonia was then introduced dropwise to the reaction mixture until a neutral pH was attained, ensuring controlled hydrolysis and homogeneous gel formation. The solution was maintained at the same temperature with constant stirring for 6 h, leading to the formation of a viscous gel. The as-formed gel was allowed to age overnight to promote network consolidation and compositional uniformity. Subsequently, the matured gel was centrifuged and repeatedly washed with distilled water, followed by 99% ethanol, to eliminate residual ions and organic impurities. The purified sample was dried in an oven for 12 h and finally calcined in a tubular furnace at 1100 °C for 4 h to remove any remaining volatile species and to achieve complete phase formation. The sequential steps of the whole procedure were followed to synthesise all the samples. The samples with 0%, 2%, 4%, 6%, and 8% Mn doping were named as MATO0%, MATO2%, MATO4%, MATO6%, and MATO8%. Finally, the samples were ground into a fine powder to prepare them for characterisation.

### Characterizations

The structural integrity and phase purity of the synthesized material were examined using X-ray diffraction (XRD), Raman spectroscopy, and Fourier transform infrared (FTIR) spectroscopy. XRD measurements were carried out using an X-ray diffractometer equipped with a Cu Kα radiation source (*λ* = 1.5406 Å). Raman spectra were recorded using a Renishaw Raman spectrometer employing an argon-ion laser with an excitation wavelength of 514.5 nm. FTIR spectroscopy was utilized to identify the characteristic vibrational modes and functional groups present in the material. The chemical composition and oxidation states of the constituent elements were investigated by X-ray photoelectron spectroscopy (XPS) using a Thermo Fisher Nexsa system with monochromatized Al Kα radiation. The acquired XPS spectra were deconvoluted using the XPSPEAK41 software. The surface morphology and microstructural features were analyzed using a field-emission scanning electron microscope (FESEM, Gemini SEM 500) operated at an accelerating voltage of 15 kV. Elemental composition and spatial distribution were determined through energy-dispersive X-ray spectroscopy (EDS) coupled with the FESEM. The optical properties of the synthesized CZTS nanoparticles were evaluated using a UV-visible spectrophotometer (PerkinElmer Lambda 365) in the wavelength range of 200–800 nm.

### Photocatalytic experiment

Tetracycline is a target pollutant used to evaluate the photocatalytic activity of MATO samples. MATO sample catalytic characteristics were measured in a simple photo-reactor using a 200 watt Xenon light. A 20 ppm pollutant solution was prepared in 100 mL of water. A UV-visible spectrophotometer was used to measure the absorbance spectra of the pollutant solution at 272 and 363 nm. Adding 20 mg of catalyst (MATO) to this solution produced the desired solution. The solution was agitated for 30 minutes in the dark to reach adsorption–desorption equilibrium with the optimal pH around 5–6 (slightly acidic). At (*t* = 0), the reaction mixture was exposed to Xe-lamp irradiation containing both UV and visible components. Considering the wide band gap of SnO_2_-based materials (>3.2 eV), the photocatalytic activity is primarily driven by the UV portion of the irradiation spectrum. Absorption data peaks were recorded every 15 minutes after *t* > 0. This experiment used samples with MATO at 0%, 4%, and 8%. The reaction rates were obtained from the slope of the graph, using the equation *k* = ln(*C*/*C*_0_)/*t*. In this context, *C* is the concentration of the pollutant at any time *t*, and *C*_0_ is the concentration at time *t* = 0.

## Results and discussion

### X-ray diffraction (XRD)

**Fig. 1:**
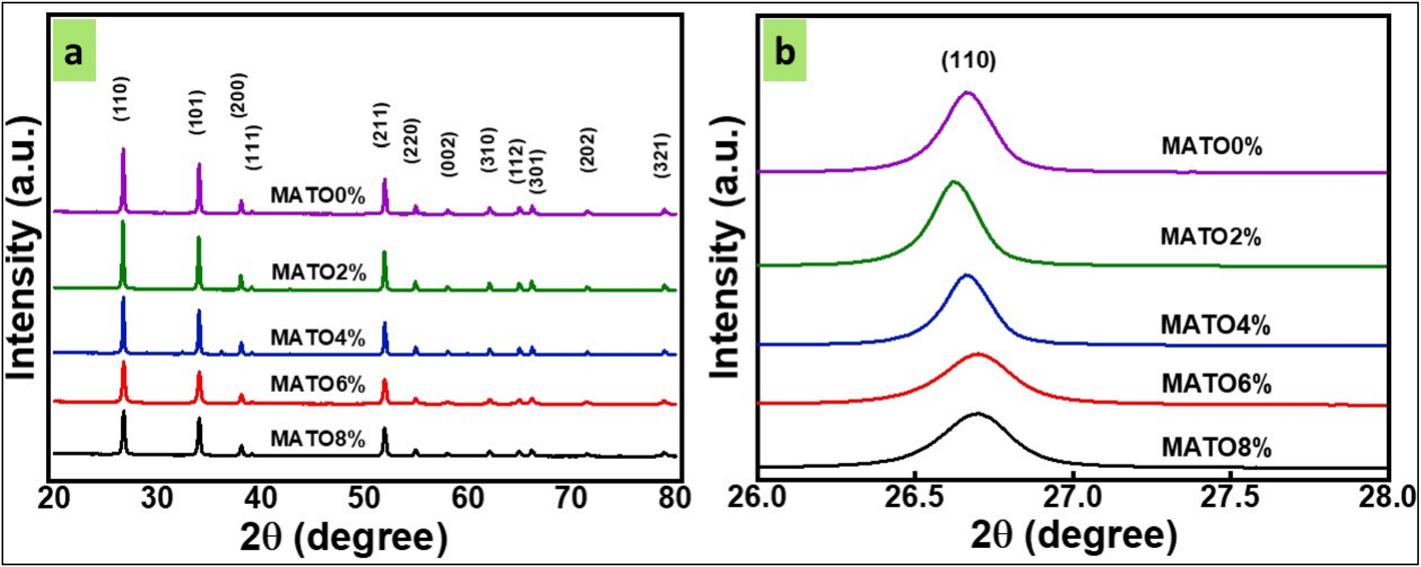
(a) X-ray diffraction of samples names as MATO (0%, 2%, 4%, 6%, and 8%). (b) Zoomed peak of (110).

**Fig. 2:**
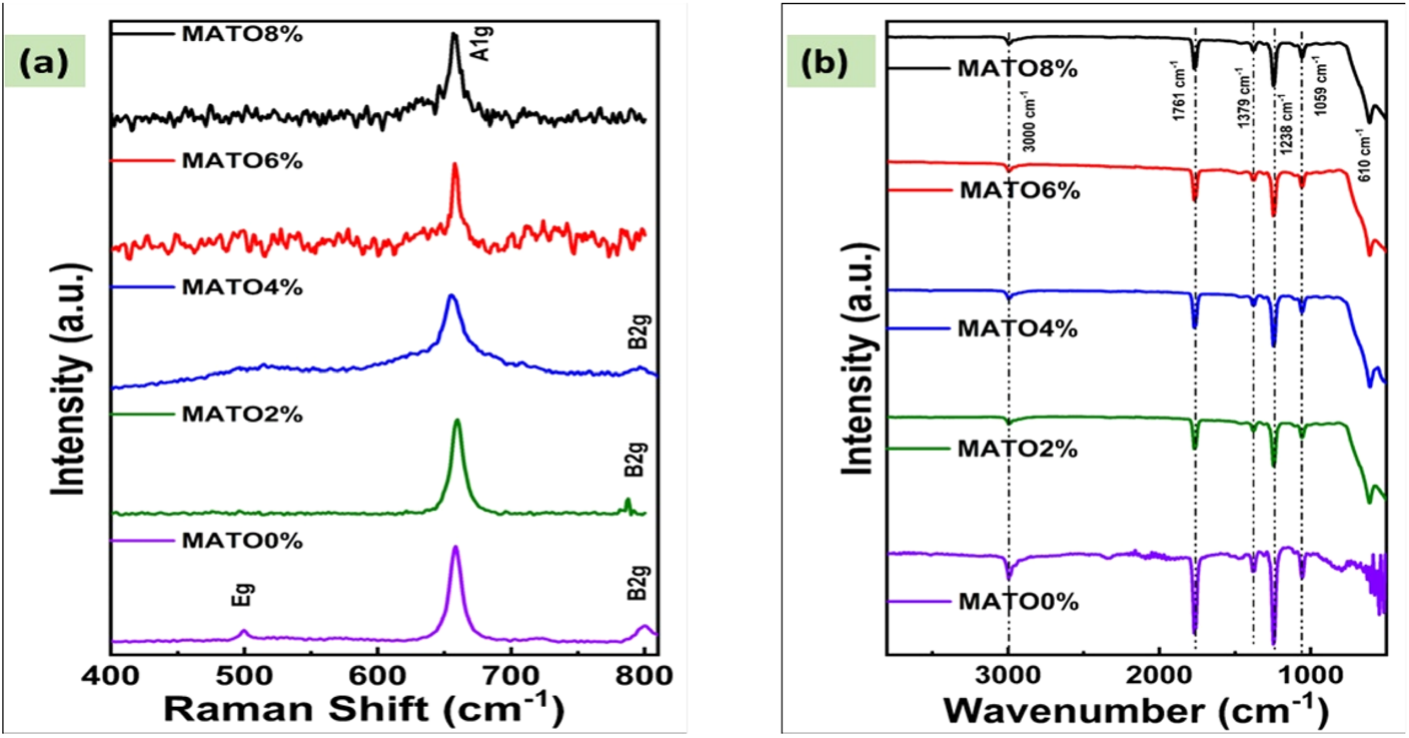
(a) Raman shift peaks of samples MATO (0%, 2%, 4%, 6%, and 8%) (b) FTIR spectrum of MATO (0%, 2%, 4%, 6%, and 8%) samples.

The crystal structure and phase evolution of the synthesized MATO samples were examined by X-ray diffraction (XRD). Fig. 1(a) presents the XRD patterns of all compositions, where the presence of well-defined and intense diffraction peaks confirms the high crystallinity and successful phase formation of the samples. From the XRD spectra, we notice the presence of main peaks assigned to planes (110), (101), (200), (111), (211), (220), (002), (310), (112), (301), (202), and (321) at diffraction angles of 26.69°, 33.99°, 38.07°, 39.05°, 51.97°, 54.89°, 58.03°, 61.91°, 64.98°, 66.09°, 71.38°, and 78.87° respectively. These planes correspond to the tetragonal rutile structure of SnO_2_ crystal, which was confirmed from JCPDS no. 77-0452.^18,19^ No extra peaks of Sb and Mn were obtained, indicating that dopants were very well incorporated into the crystal. The diffraction peak (110) of MATO2% shifted slightly towards a lower angle as the Mn doping content increased from 0% to 2%. Then, the peak shifted towards higher angles for 4%, 6%, and 8% Mn doping, as shown in Fig. 2(b). Additionally, the broadening of the peaks increased as the doping percentage of Mn was increased from 0% to 8%, as shown in Fig. 1(b). In the meantime, the intensity of the diffraction peaks decreases as the percentage of doping increases, indicating that Mn has substituted for Sn in the SnO_2_ host. This shift and broadening of the most intense peak indicate doping of Mn in the SnO_2_ matrix. Further, the effect of the shift and broadening of all the peaks can be reflected by calculating the crystallite size.

The decreases in crystallite size, lattice parameters and cell volume can be attributed to the substitution of small-size Mn^4+^ ions (0.53 Å) in place of Sn^4+^ ions (0.69 Å).^13^

The obtained calculated lattice parameters *a*, *b*, *c*, and volume *v* are given below in Table 1.

**Table 1:** The table presents the lattice parameters *a*, *b*, *c*, and volume *v* of the synthesised MATO series

Sample name	MATO0%	MATO2%	MATO4%	MATO6%	MATO8%
*a* = *b* (nm)	0.4728	0.4726	0.4725	0.4720	0.4710
*c* (nm)	0.3178	0.3178	0.3177	0.3176	0.3176
*v* (nm)^3^	0.07104	0.0710	0.0709	0.0707	0.0704

### Raman spectroscopy

The phase structure of the Mn–Sb-doped SnO_2_ samples underwent additional evaluation through the application of Raman Spectroscopy as shown in Fig. 2(A). The samples were stimulated at an excitation energy of 532 cm^−1^, revealing distinct modes of B2g, Eg, and A1g at both lower and zero Mn doping levels. As the doping percentage of Mn was adjusted to 6% and 8%, the A1g peak emerged distinctly, whereas the other peaks remained obscured (Fig. 2). The A1g mode was detected at approximately 656 cm^−1^ across all samples and is regarded as a distinctive indicator for the emergence of the rutile phase within the SnO_2_ matrix,^20^ the B2g mode was obtained around 790 cm^−1^ in the MATO0%, MATO2%, and MATO4% samples, and the Eg mode at 500 cm^−1^ in the MATO0% sample; these modes also correspond to the tetragonal phase formation of doped SnO_2_ samples.^21^ B_2_g peak is well pronounced in samples with (0–4)% Mn doping concentration, with slight broadening in MATO4%, indicating lattice distortion and defective state, while retaining the rutile structure.^22^ For MATO6% and MATO8%, the B_2_g peak disappears, which is consistent with the structural reconstruction and growth of a Mn-rich defective environment in the SnO_2_ lattice.^22,23^

### FTIR analysis

To identify the functional group, an additional vibrational analysis using Fourier Transform Infrared Spectroscopy (FTIR) was performed. FTIR of all samples were obtained within the wavenumber range of (4000–400) cm^−1^ as shown in Fig. 2(B). The O–Sn–O lattice stretches, as evidenced by the metal–oxygen lattice stretching wave modes, are matched by the strong peaks observed within 567–636 cm^−1^. The observation of bands in the spectrum around 600 cm^−1^ suggests the formation of a metal oxide composite, situated within the fingerprint region of M–O binding.^24^ The peak observed at 1059 cm^−1^ shows C–N stretching.^25^ Three prominent peaks are observed at 1238 cm^−1^, 1379 cm^−1^, and 1761 cm^−1^, which are correlated to the C–O stretch, O–H bending and C–N stretching modes, respectively.^26^ The extensive band observed at 3000 cm^−1^ corresponds to the stretching vibrations of hydroxyl groups associated with O–H bonds.^27^ The existence of the above-defined bands confirmed the formation of SnO_2_ and doped SnO_2_ nanoparticles.^25^ The phase purity, as evidenced by XRD, Raman, and FTIR, provides constructive proof that the SnO_2_ prepared samples can be tested for photocatalytic applications.

### FESEM and EDS spectroscopy

**Fig. 3:**
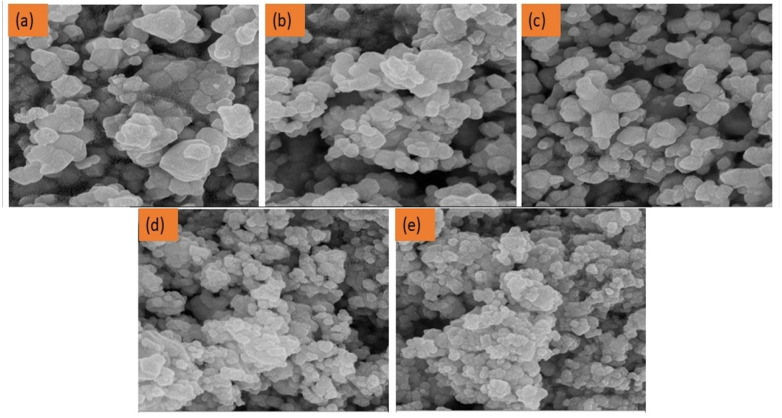
FESEM images of MATO samples with (a) MATO0%, (b) MATO2%, (c) MATO4%, (d) MATO6%, (e) MATO8%.

The morphological structure of the synthesised series was analysed using FESEM. Fig. 3(a–e) presents the images of the samples exhibiting different Mn concentrations. The pictures demonstrate a variety of polygons, including hexagons, pentagons, and almost spherical arrangements, along with some irregular grain patterns. All the samples show very similar morphological traits. However, as the concentration of Mn went up, the size of the grains went down. When the grain size gets smaller, the surface-to-volume ratio gets bigger. This might make photocatalysis better by giving it more active sites. The grain size decreases regularly from 78 nm to 36.8 nm for MATO0% and MATO8%, respectively (Fig. 5). The decrease in grain size complemented the trend of decreasing crystallite size. Additionally, the decline in grain size may impact the application in which the sample is exposed to light, such as photocatalysis and photodetection.

**Fig. 4:**
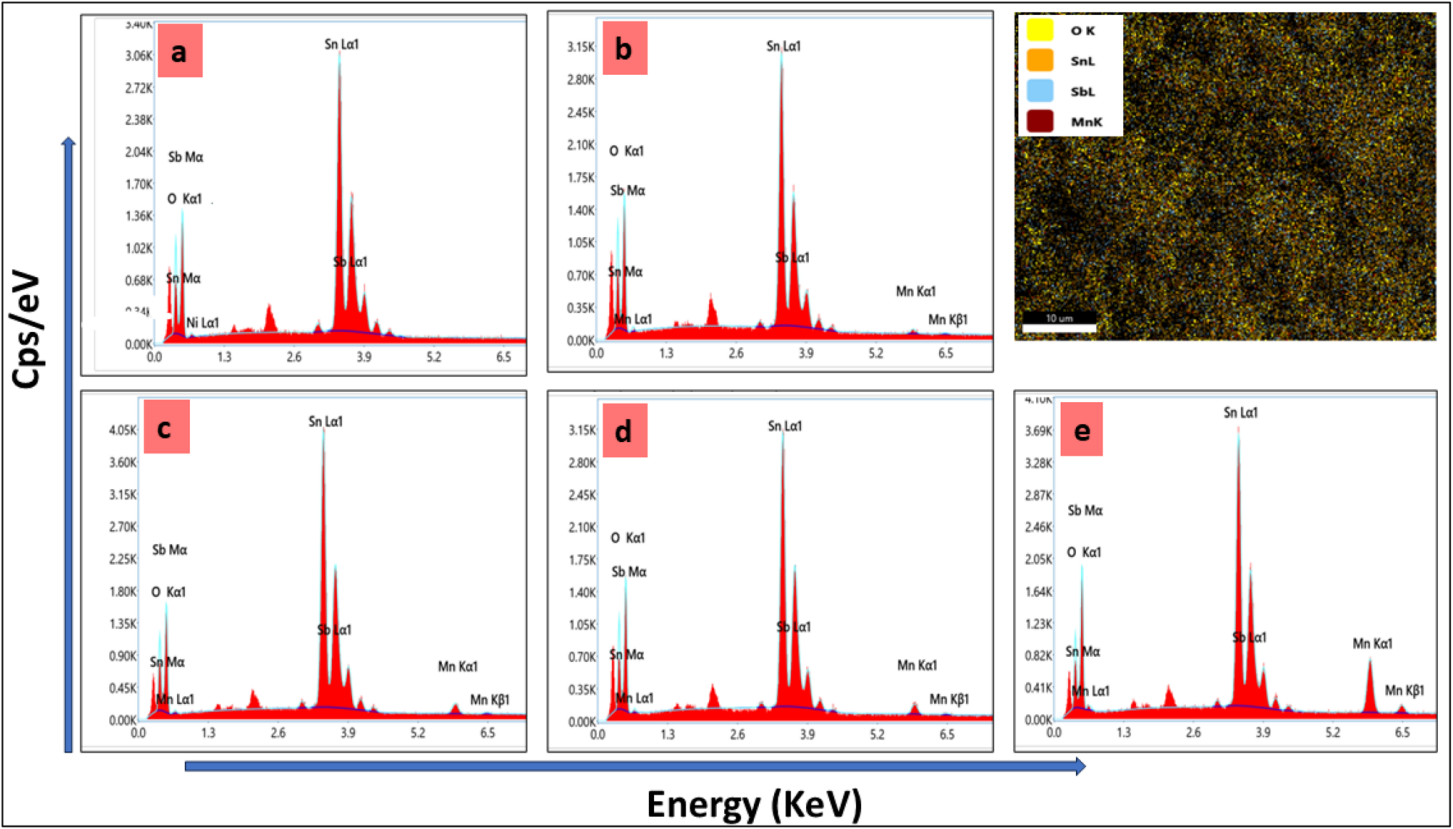
EDS plots of all the Mn/Sb-doped SnO_2_ samples (a) MATO0%, (b) MATO2%, (c) MATO4%, (d) MATO6%, (e) MATO8%.

The electron dispersive spectroscopy presented in Fig. 4 shows the elemental makeup of the elements present in the MATO series. It can be clearly seen in Fig. 4(a–e) that peaks corresponding to Sn, Sb, and O are present. However, the Mn peak is present in Fig. 4(b–e), with its peak intensity increasing, which is consistent with the Mn doping during synthesis and the elemental composition by weight and atomic percentage has been tabulated in Table 1S.

**Fig. 5:**
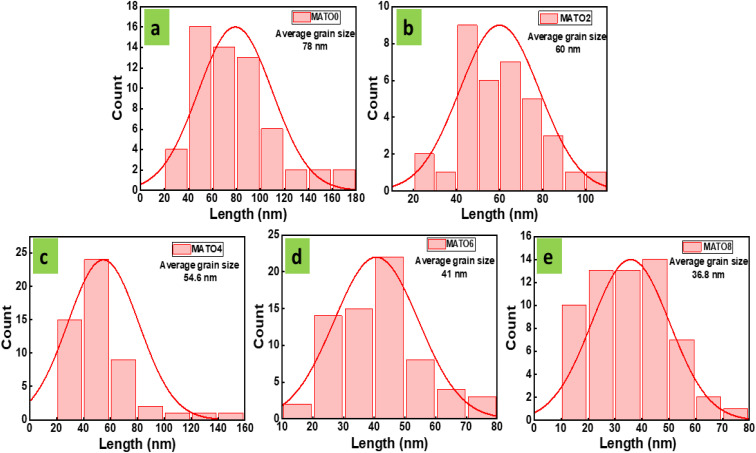
Grain size histograms of MATO samples with (a) MATO0%, (b) MATO2%, (c) MATO4%, (d) MATO6%, (e) MATO8%.

### Optical spectroscopy

**Fig. 6:**
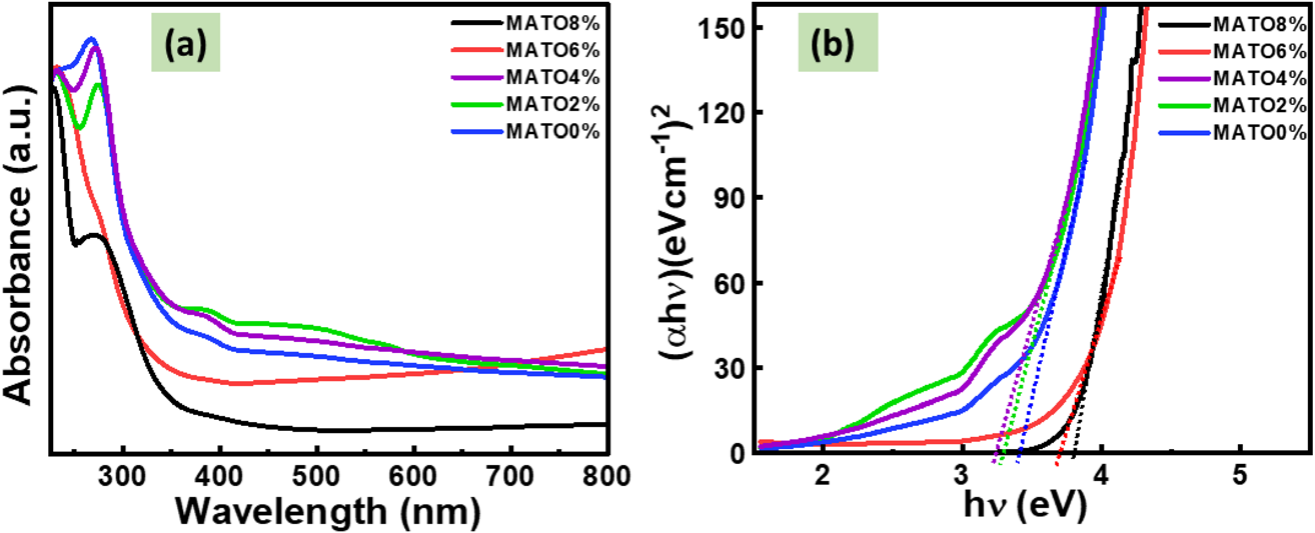
(a) UV visible absorption spectrum of MATO samples and (b) Tauc plot of MATO samples for band gap calculation.

Fig. 6(a) illustrates the UV-visible absorption spectra of the synthesized MATO series. All samples exhibit a similar absorption profile in the ultraviolet region, indicating comparable electronic transitions and optical behaviour across the series. The optical band gap energies were estimated using Tauc analysis, as shown in Fig. 6(b). The Tauc plots were constructed from the absorbance data by examining the relationship between the optical absorption coefficient (*α*) and the photon energy (*hν*). The optical band gap (*E*_g_) was determined using the Tauc relation:

1 *αhν*^*n*^ = *A*(*hν* − *E*_g_)

In this context, *ν* is the frequency of incident light, *h* is the Planck constant, and *n* is 1/2 for the direct allowed transition, as is the case in the present instance. The optical band gap, *E*_g_, was determined using the straight-line portion of the (*αhν*)^2^*vs. hν* curve at *α* = 0. The optical bandgap values for MATO0%, MATO2%, and MATO4% are, respectively, 3.37 eV, 3.29 eV, and 3.24 eV, which are nearly equal. However, they decrease slightly with an increase in the Mn doping percentage from 0% to 4%. The reduction in band gap of SnO_2_ with lower Mn doping can result from the presence of holes.^28^ In general, Mn substitution generates two-hole states associated with the O 2p orbitals, leading to the emergence of an impurity band within the forbidden gap. Upon excitation, valence-band electrons interact with these impurity states before transitioning toward the conduction band, thereby reducing the effective band gap through impurity-band-mediated electronic transitions.^29^ For the samples MATO6% and MATO8% the band gap values are 3.71 eV and 3.80 eV, respectively. At elevated doping concentrations, the band gap began to widen due to the presence of Mn interstitials, which emerged from the limited solubility of Mn in SnO_2_. The increase in the optical band gap is mainly attributed to the Burstein–Moss shift, caused by Mn-induced carrier doping that elevates the Fermi level above the conduction band minimum. This state filling suppresses low-energy optical transitions, leading to an apparent band-gap widening.^28^ The observed increase in the band gap upon Mn doping can be attributed to the introduction of additional electronic states within the band gap of SnO_2_, arising from the incorporation of Mn^4+^ ions. These localized states facilitate charge–transfer transitions between the valence and conduction bands of SnO_2_ and the Mn dopant levels, thereby modifying the optical transition pathways. Similar behaviours has been reported by Venugopal *etal.*,^30^ for Mn-doped SnO_2_ synthesized *via* the precipitation method, further corroborating the present observations.

### XPS spectroscopy

**Fig. 7:**
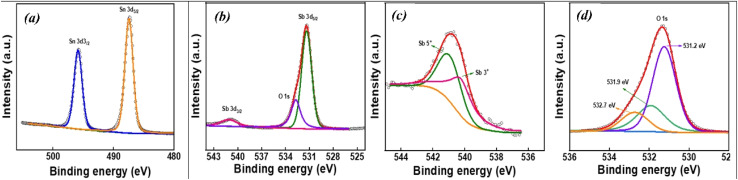
(a) XPS of Sn, (b) XPS of Sb, (c) deconvoluted Sb 3d_3/2_ and (d) shows O 1s spectra of ATO.

**Fig. 8:**
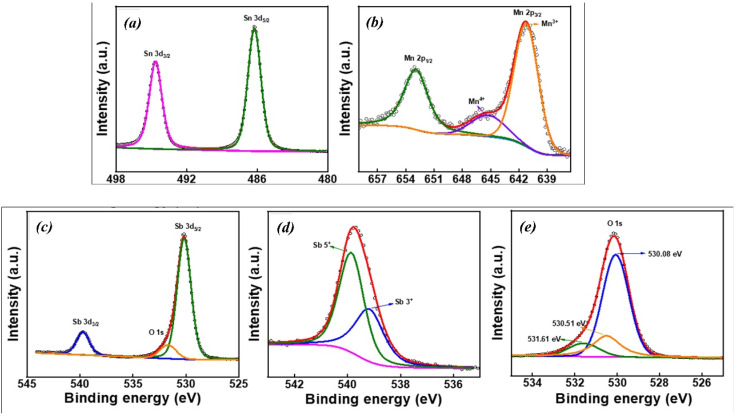
(a) XPS of Sn, (b) XPS of Mn, (c) XPS of Sb, (d) deconvoluted Sb 3d_3/2_, and (e) shows O 1s spectra of MATO.

The X-ray photoelectron spectroscopy (XPS) spectra of Sb-doped SnO_2_ (ATO) and Mn–Sb co-doped SnO_2_ (MATO) samples are shown in Fig. 7 and 8, respectively. The corresponding survey spectra of the ATO and MATO samples are presented in Fig. 7(a) and 8(a), confirming the presence of all constituent elements without detectable impurity peaks. All binding energies were calibrated using the adventitious carbon C 1s peak positioned at 284.6 eV. Fig. 7(b) and 8(b) display the high-resolution Sn 3d core-level spectra, which exhibit two well-resolved peaks attributed to Sn 3d_5/2_ and Sn 3d_3/2_ The Sn 3d_5/2_ peaks appear at binding energies of approximately 486.6 eV for ATO and 486.3 eV for MATO, while the Sn 3d_3_/_2_ peaks are observed at 495.3 eV and 495.7 eV for the respective samples. The measured spin–orbit splitting of ∼8.5 eV between the Sn 3d_3/2_ and Sn 3d_5/2_ peaks is consistent with reported values for Sn^4+^, confirming the +4 oxidation state of tin in the SnO_2_ lattice.^6^ Through the application of the XPSPEAK 41 programme, the O1S spectra for SnO_2_ reveal three distinct peaks located at 531.2 eV, 531.9 eV, and 532.7 eV (Fig. 7(e)) for ATO and 530.08 eV, 530.51 eV, and 531.61 eV (Fig. 8(f)) for MATO. The peaks underwent deconvolution and were fitted using both Gaussian and Lorentzian functions. The presence of lattice oxygen (O^2−^) associated with Sn^4+^ and the O^2−^ ions in oxygen-deficient regions is responsible for the two observed peaks at 530.08 eV and 530.51 eV, respectively, in MATO, and the binding energy component for the adsorbed oxygen species was determined to be 531.61 eV. Altering the ratio of oxygen-rich to oxygen-poor states explains the chemical transformation observed with increased doping. Electrons can transition between different oxygen vacancies due to the significant alterations in electrical properties resulting from oxygen deficiency.^31^ The evidence for Sb doping in SnO_2_ is demonstrated by the overlap of the O 1s and Sb-3d peaks, as illustrated in Fig. 7(c) and 8(d) for ATO and MATO, respectively. The differentiation of the O 1s and Sb-3d peaks was achieved through the fitting of the experimental data, as illustrated in Fig. 7 and 8. The surface antimony exhibits a restricted range of identifiable oxidation states. The binding energies of 3d_5/2_ (530.51 eV for ATO and 529.6 eV for MATO) and 3d_3/2_ (541.04 eV for ATO and 539.8 eV for MATO) align well with the published values for the Sb^5+^ state.^32^Fig. 8(c) displays the deconvoluted Mn 2p core-level XPS spectrum of the MATO sample. The peaks centered at binding energies of approximately 641.5 eV and 653.7 eV are assigned to the spin–orbit–split Mn 2p_3/2_ and Mn 2p_1/2_ components, respectively, indicating the coexistence of Mn^3+^ and Mn^4+^ oxidation states.^33^ In addition, the feature observed at around 646.1 eV is attributed to satellite contributions associated predominantly with the Mn^4+^ valence state.^34^ The presence of these characteristic peaks confirms the successful incorporation of Mn ions into the SnO_2_ lattice and verifies the mixed valence states of Mn in the co-doped system. The XPS O 1s spectra indicate the presence of oxygen vacancies (*V*_O_) in the synthesized material. Oxygen vacancies are intrinsic point defects that can form during synthesis due to lattice distortion or partial oxygen deficiency.^35^ The formation of *V*_O_ introduces localized defect states within the bandgap, which can effectively trap electrons and facilitate charge transfer processes.^36^ These defect sites promote the separation of photogenerated electron–hole pairs and suppress their recombination, thereby enhancing photocatalytic efficiency. In addition, oxygen vacancies serve as active adsorption centres for reactant molecules and oxygen species, which can accelerate surface redox reactions during photocatalysis. Consequently, the presence of *V*_O_ contributes to improved charge carrier mobility and increased catalytic activity of the semiconductor system.^37^ The deconvoluted O 1s spectra were quantitatively analyzed to estimate the relative concentration of oxygen vacancies. The O 1s peak was resolved into three components corresponding to lattice oxygen (*O*_L_), oxygen associated with vacancy-related defect sites (*O*_V_), and surface adsorbed oxygen species (*O*_A_). The relative proportion of oxygen vacancies was calculated using the integrated peak area ratio 
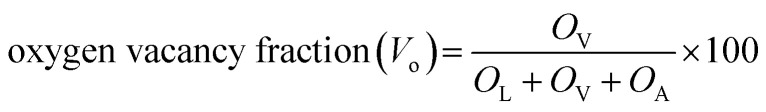
. The analysis reveals that the Mn–Sb co-doped SnO_2_ sample possesses a higher fraction of defect-related oxygen species, indicating an increased concentration of oxygen vacancies. The presence of a higher vacancy density is expected to facilitate charge carrier separation and enhance surface catalytic reactions, thereby contributing to improved photocatalytic degradation efficiency.^38^ The calculated vacancy fraction is approximately 22% for the ATO sample and 25% for the Mn–Sb co-doped MATO sample, indicating that Mn incorporation increases the concentration of oxygen vacancy defects, which can enhance charge separation and catalytic activity.

### BET analysis

**Fig. 9:**
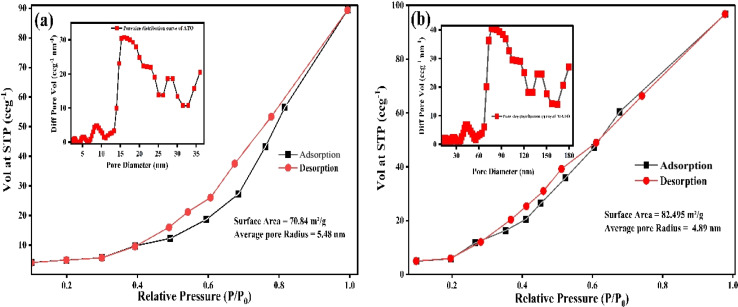
BET analysis (a) ATO and (b) Mn–Sb co-doped MATO (4%).

The textural properties of the ATO and Mn–Sb co-doped MATO (4%) samples were investigated using nitrogen adsorption–desorption measurements, and the corresponding isotherms along with the BJH pore size distributions are presented in Fig. 9(a) and (b). Both samples exhibit a type IV adsorption–desorption isotherm with a noticeable hysteresis loop at intermediate relative pressures (*P*/*P*_0_ ≈ 0.4–1.0), which is characteristic of mesoporous materials formed by interconnected pore networks. Such hysteresis behavior is commonly attributed to capillary condensation within mesopores and indicates the presence of a well-developed porous structure. Similar adsorption–desorption behavior has been reported for SnO_2_-based nanostructures with mesoporous architectures.^39^ From the BET analysis, the specific surface area of the ATO sample is calculated to be 70.84 m^2^ g^−1^, with an average pore radius of 5.48 nm, indicating the formation of mesoporous structures. In comparison, the MATO (4%) sample shows a higher specific surface area of 82.495 m^2^ g^−1^ and an average pore radius of 4.89 nm, suggesting that Mn incorporation modifies the microstructure and leads to an increase in surface area with slightly reduced pore dimensions as tabulated in Table 2S. The BJH pore size distribution curves further confirm that both samples possess mesopores predominantly in the range of approximately 15–35 nm for ATO and 60–150 nm for MATO (4%), indicating a hierarchical pore structure.^40^

The increased surface area observed in the MATO (4%) sample may be attributed to structural distortion and defect formation induced by Mn doping, which can inhibit particle growth and promote the formation of additional porous channels. It is well known that the gas adsorption capacity and catalytic performance of SnO_2_-based materials are strongly influenced by their surface area and porosity. A higher surface area provides more active adsorption sites for reactant molecules and facilitates faster diffusion of gases within the porous network.^41^ Consequently, the enhanced surface area and optimized pore structure of the MATO (4%) sample are expected to improve its catalytic and sensing performance. Similar relationships between surface area, porosity, and sensing efficiency have been reported for SnO_2_ nanomaterials in gas sensing applications. The BET results demonstrate that Mn incorporation significantly modifies the microstructure of SnO_2_, leading to improved porosity and surface area, which are beneficial for enhancing surface reactions and mass transport processes during photocatalytic or sensing applications.^42^

### Photocatalytic degradation

**Fig. 10:**
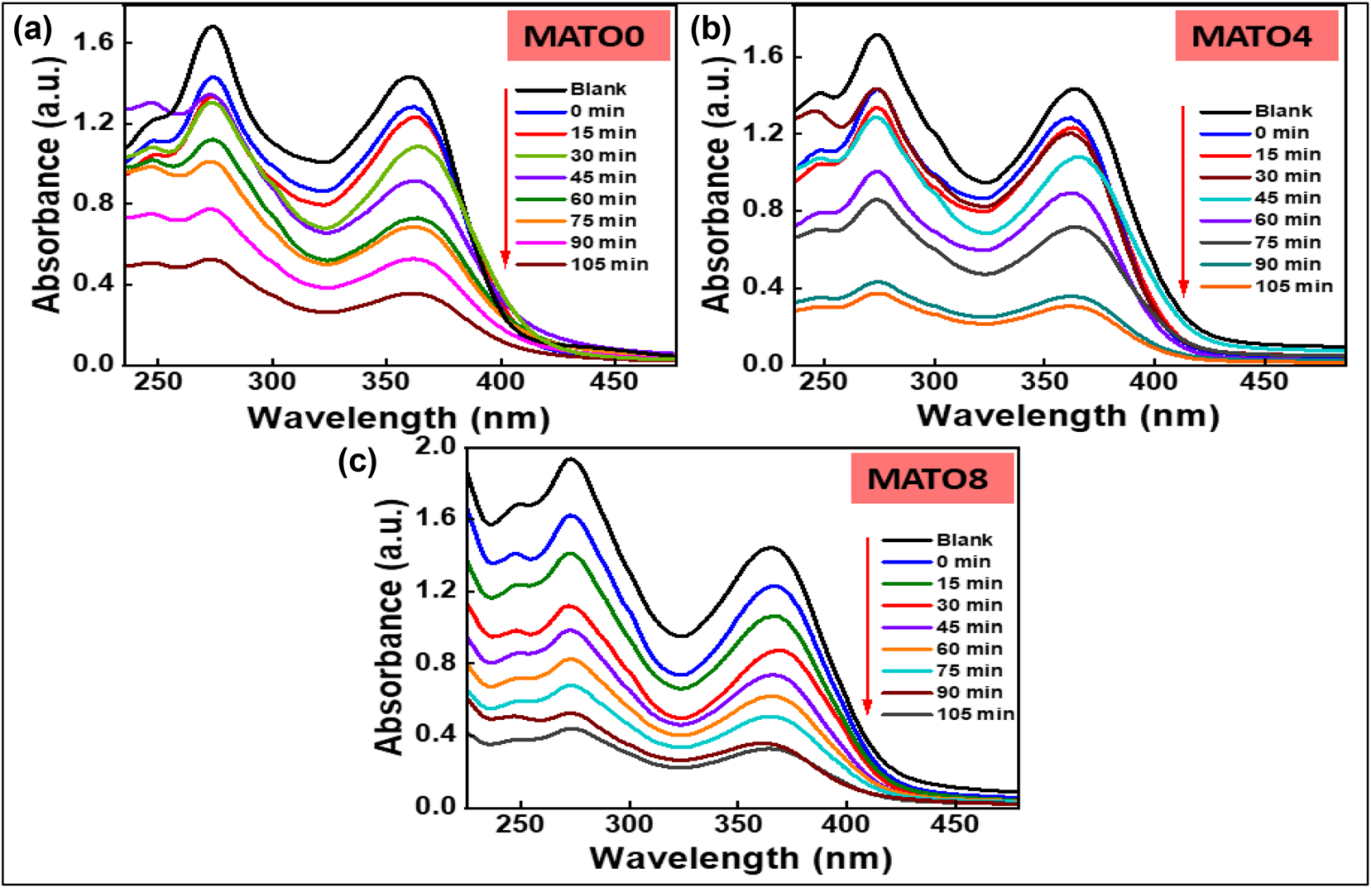
(a) Absorbance spectrum of tetracycline acetate pollutant when treated under light irradiation with MATO0%, (b) MATO4% and (c) MATO8%.

Tetracycline serves as a target pollutant from the pharmaceutical industry to assess the photocatalytic effectiveness of MATO samples. The photocatalytic activity of the MATO samples was evaluated using a custom-designed photoreactor equipped with a 200 W xenon lamp as the light source. A model pollutant solution with a concentration of 20 ppm was prepared by dissolving the pollutant in 100 mL of deionized water. The characteristic absorbance peaks of the pollutant, located at 272 nm and 363 nm, were monitored using a UV-visible spectrophotometer. For each experiment, 20 mg of the MATO catalyst was dispersed into the pollutant solution under continuous stirring. Prior to light irradiation, the suspension was magnetically stirred in the dark for 30 min to establish adsorption–desorption equilibrium between the catalyst surface and pollutant molecules. Subsequently, at *t* = 0, the reaction mixture was exposed to ultraviolet (UV) irradiation under continuous stirring, and the initial absorption spectrum was recorded. After irradiation (*t* > 0), aliquots were withdrawn at regular intervals of 15 min, and the corresponding absorption spectra were measured to monitor the photocatalytic degradation process. This experiment was done for MATO0%, MATO4%, and MATO8% samples. Fig. 10 displays the absorbance spectrum of the tetracycline pollutant using MATO0%, MATO4%, and MATO8% samples as catalysts.

The absorption peaks of the pollutant were observed at 272 nm and 363 nm. From Fig. 10, it can be observed that the peak intensity decreases with increasing light irradiation time, indicating a decline in contaminant concentration, which is consistent with the Beer–Lambert law. This decline in intensity is due to the photocatalytic degradation of the pollutant using the catalyst. The percentage degradation can be presented using the equation:2
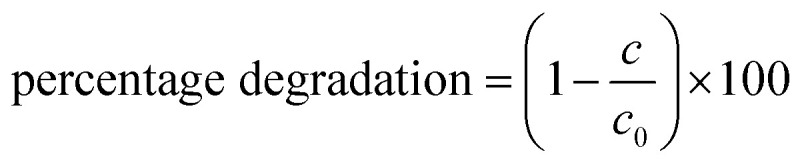
*C*_0_ indicates the initial absorption at time *t* equals zero, whereas *C* represents the absorbance at time *t* following the introduction of the catalyst.

The results show that tetracycline was efficiently degraded using MATO0%, MATO4%, and MATO8% as catalysts. The degradation percentage of the tetracycline pollutant using MATO0, MATO4, and MATO8 samples, respectively, is 72.6%, 79.5%, and 73.2%. The degradation percentage of the pollutant using MATO4% is maximum in the present case. One of the main reasons is the optimal doping percentage of Mn. After a limited doping percentage, such as 4%, the agglomeration begins to increase, thereby reducing the active sites. This reduction in active sites is a key reason for the decline in percentage degradation, as in the present case, using MATO8.^43^ Also, band gap reduction is a crucial parameter that determines the enhanced degradation efficiency of a catalyst.^44^ In the present case, the band gaps are approximately 3.37 eV for MATO0% and 3.21 eV for MATO4%, but for MATO8%, the band gap increases to 3.80 eV. The band gap values and agglomeration are increased for MATO8, which are the primary reasons for the decline in degradation percentage compared to MATO4.

**Fig. 11:**
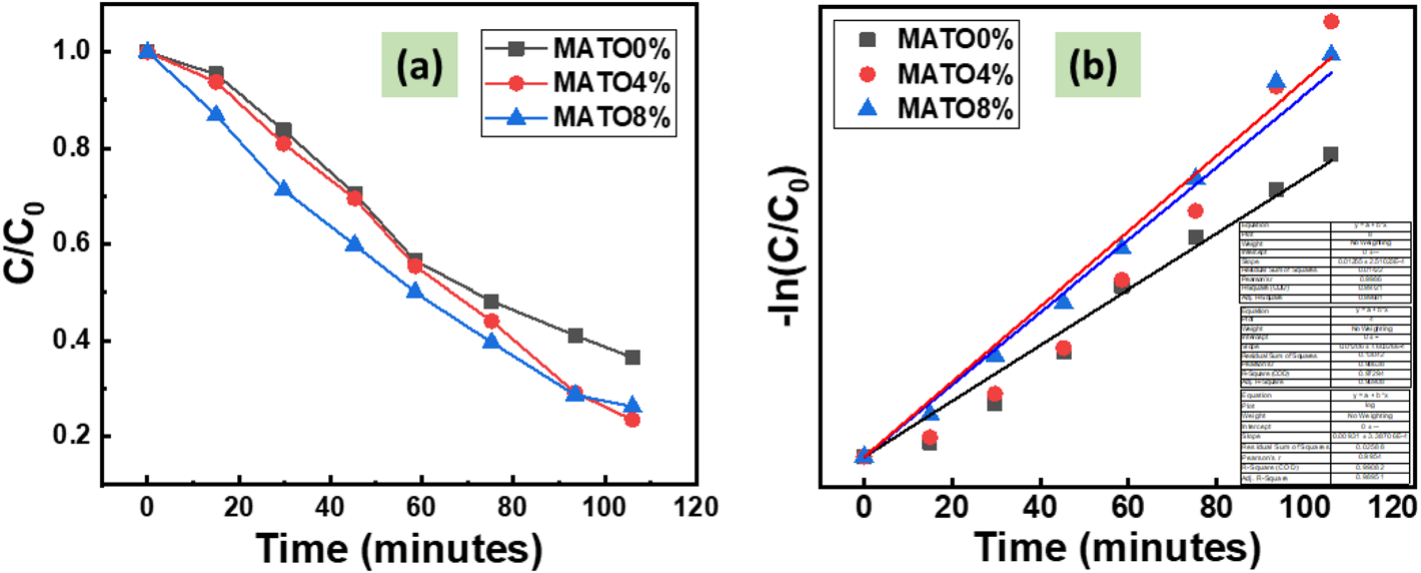
The photocatalytic degradation of pollutant solution (*C*/*C*_0_) is examined as a function of irradiation time using the MATO catalyst (a) a kinetics plot displaying ln(*C*/*C*_0_) as a function of illumination time (min) (b).

The degradation curves of the pollutant solution are shown in Fig. 11. The reaction's designation as a pseudo-first-order reaction is supported by the linear relationship seen in the Fig. 11(a). The rate constants obtained for MATO0%, MATO4%, and MATO8% are respectively 0.00931 min^−1^, 0.0125 min^−1^ and 0.0120 min^−1^. The reaction rates were obtained from the slope of the graph Fig. 11(b), using the equation given below.3
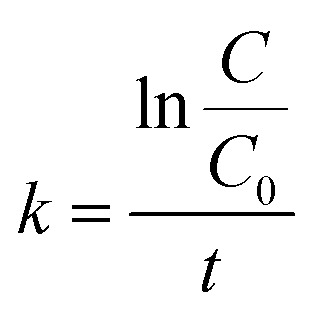


**Fig. 12:**
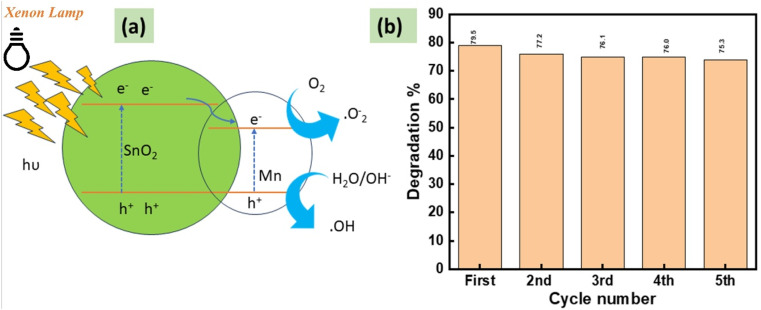
(a) Schematic of the MATO4% photocatalytic mechanism for the tetracycline degradation. (b) Histogram plot defining degradation% for different cycles.

Pure SnO_2_ generates electron–hole pairs primarily when exposed to ultraviolet (UV) irradiation due to its wide band gap. The photogenerated electrons can react with dissolved oxygen to produce superoxide radicals (˙O_2_^−^), while holes may oxidize water molecules to produce hydroxyl radicals (˙OH). These reactive oxygen species are commonly reported to participate in the degradation of organic pollutants in SnO_2_-based photocatalytic systems. The schematic mechanism shown in Fig. 12(a) therefore represents a proposed pathway based on established photocatalytic processes in similar metal-oxide systems.^28,44^Fig. 12(b) presents the photocatalytic activity of repeated five cycles with a good retention efficiency. This reusability test validates that the materials have potential for pollution degradation.

To evaluate the photocatalytic efficiency of the Mn–Sb co-doped SnO_2_ samples, a comparison with recently reported SnO_2_-based photocatalysts is presented in Table 2. The comparison includes parameters such as catalyst dosage, pollutant concentration, light source, degradation efficiency, and kinetic rate constant. It can be observed that several strategies, including defect engineering, heterojunction formation, and metal doping, have been employed to enhance photocatalytic activity in SnO_2_-based systems. Although some heterostructure systems exhibit higher degradation efficiencies, the Mn–Sb co-doped SnO_2_ synthesized in this work demonstrates competitive photocatalytic performance with a degradation efficiency of 79.5% and a rate constant of 0.0125 min^−1^ under Xe lamp irradiation. The improved activity can be attributed to Mn-induced defect states and enhanced charge separation in the SnO_2_ lattice.

**Table 2:** Comparison of photocatalytic degradation performance of SnO_2_-based photocatalysts reported in recent literature

Photocatalyst	Pollutant	Catalyst dose	Pollutant conc	Light source	Degradation efficiency	Rate constant (min^−1^)	Ref.
Mn–Sb co-doped SnO_2_ (this work)	Tetracycline	20 mg/100 mL	20 ppm	Xe lamp (200 W)	79.5%	0.0125	This work
Mo-modified SnO_2_ QDs	Antibiotic pollutants	20 mg/50 mL	10 mg L^−1^	Visible light	92%	0.018	7
Oxygen-deficient SnO_2_ QDs	Oil pollutants	25 mg/100 mL	20 mg L^−1^	Visible light	90%	0.016	14
Mn-doped SnO_2_ nanoparticles	Congo red dye	30 mg/100 mL	10 ppm	UV light	85%	0.014	28
g-C_3_N_4_/Zn_2_SnO_4_ heterojunction	Tetracycline	20 mg/50 mL	10 mg L^−1^	Xe lamp	95%	0.20	36

### Scavenging experiment

**Fig. 13:**
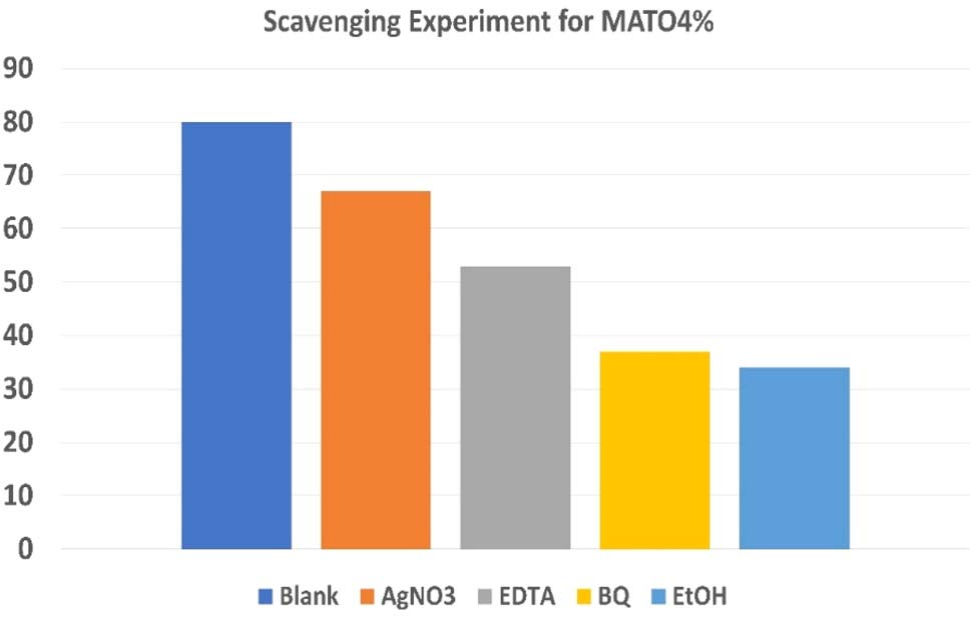
Scavenging experiment of MATO4%.

To elucidate the dominant reactive species involved in the photocatalytic degradation process, scavenger trapping experiments were performed, and the results are presented in Fig. 13. Specific scavengers were employed to selectively quench different active species: AgNO_3_ (e^−^ scavenger), EDTA (h^+^ scavenger), benzoquinone (BQ, ˙O_2_^−^ scavenger), and ethanol (EtOH, ˙OH scavenger). In the absence of scavengers (blank), the degradation efficiency of MATO4% was approximately 80%. Upon the addition of scavengers, a systematic decrease in degradation efficiency was observed. The presence of AgNO_3_ reduced the degradation efficiency to ∼67%, indicating that photogenerated electrons contribute to the reaction. Similarly, the addition of EDTA decreased the efficiency to ∼53%, confirming the role of holes. A more significant suppression was observed in the presence of BQ (∼37%) and EtOH (∼34%), suggesting that superoxide radicals (˙O_2_^−^) and hydroxyl radicals (˙OH) are the dominant reactive species governing the degradation process. Based on these observations, a plausible photocatalytic mechanism is proposed as follows. Upon light irradiation, the photocatalyst generates electron–hole pairs:

4 SnO_2_ + *hν* → SnO_2_(e^−^) + h^+^

The photogenerated electrons can be effectively trapped or transferred *via* Mn species, enhancing charge separation:

5 SnO_2_(e^−^) + Mn → SnO_2_ + Mn(e^−^)

These electrons subsequently react with dissolved oxygen to produce superoxide radicals:

6 SnO_2_(e^−^) + O_2_ → O_2_˙^−^ + SnO_2_

Meanwhile, photogenerated holes participate in oxidation reactions involving water molecules:

7 Mn(h^+^) + H_2_O → Mn + OH˙ + h^+^

The generated superoxide radicals further react with water to produce hydroperoxyl radicals and hydroxyl species:

8 O_2_˙^−^ + H_2_O → HO_2_˙^−^ + OH˙

Hydroxyl radicals can also combine to form hydrogen peroxide:

9 OH˙ + OH˙ → H_2_O_2_

Finally, these highly reactive oxygen species (˙O_2_^−^ and ˙OH) attack and degrade the organic pollutants into harmless products:

10 O_2_˙^−^ + OH˙ + drug solution → degradation products

## Conclusion

In summary, Mn–Sb co-doped SnO_2_ nanoparticles were successfully synthesized *via* a sol–gel method and systematically investigated for their structural, optical, and photocatalytic properties. XRD and Raman analyses confirmed the formation of a single-phase tetragonal rutile structure, demonstrating the successful incorporation of Mn and Sb into the SnO_2_ lattice without secondary phases. The crystallite size decreased from 36.55 nm to 24.54 nm with increasing Mn concentration, indicating lattice distortion induced by dopant substitution. FESEM observations revealed reduced grain size and morphological variations that can contribute to increased active surface sites. Optical analysis showed band-gap modulation from 3.37 to 3.24 eV at moderate Mn doping, while higher doping levels caused band-gap widening due to the Burstein–Moss effect. BET measurements revealed that Mn incorporation enhanced the specific surface area from 70.84 to 82.49 m^2^ g^−1^ and generated mesoporous structures favorable for catalytic reactions. XPS analysis further confirmed the presence of mixed valence states and an increased concentration of oxygen vacancies (∼25%), which play a key role in facilitating charge separation and improving photocatalytic activity. Photocatalytic experiments demonstrated that the optimally doped MATO4% sample exhibited the highest tetracycline degradation efficiency of 79.5% with a rate constant of 0.0125 min^−1^ under Xe-lamp irradiation. The improved catalytic performance is attributed to the synergistic effects of band-gap modulation, increased surface area, and defect-induced charge separation. These findings highlight Mn–Sb co-doped SnO_2_ as a promising photocatalyst for antibiotic pollutant remediation. Future studies may focus on constructing heterostructures or defect-engineered composites to further enhance visible–light activity and practical wastewater treatment efficiency.

## Conflicts of interest

The author's known competing financial interests and personal connections had no bearing on the material presented in this article.

## Data Availability

All the data generated or analyzed during this inquiry is included in this paper.

## References

[cit1] Lwin H. M., Zhan W., Song S., Jia F., Zhou J. (2019). Visible-light photocatalytic degradation pathway of tetracycline hydrochloride with cubic structured ZnO/SnO_2_ heterojunction nanocatalyst. Chem. Phys. Lett..

[cit2] Li C., Awasthi M. K., Liu J., Yao T. (2025). Veterinary tetracycline residues: Environmental occurrence, ecotoxicity, and degradation mechanism. Environ. Res..

[cit3] Wang S. (2017). *et al.*, Tetracycline resistance genes identified from distinct soil environments in China by functional metagenomics. Front. Microbiol..

[cit4] Shao S., Wu X. (2020). Microbial degradation of tetracycline in the aquatic environment: a review. Crit. Rev. Biotechnol..

[cit5] Wu D., Dai S., Feng H., Karunaratne S. H. P. P., Yang M., Zhang Y. (2024). Persistence and potential risks of tetracyclines and their transformation products in two typical different animal manure composting treatments. Environ. Pollut..

[cit6] Ahmad T., Ansari M. Z. (2023). Structural and optical characteristics of Sb doped SnO_2_ nanoparticles and their boosted photocatalytic activity under visible light irradiation. Ceram. Int..

[cit7] Wang Y. (2022). *et al.*, Mo-modified band structure and enhanced photocatalytic properties of tin oxide quantum dots for visible-light driven degradation of antibiotic contaminants. J. Environ. Chem. Eng..

[cit8] Medhi R. (2019). *et al.*, Uniformly Spherical and Monodisperse Antimony- and Zinc-Doped Tin Oxide Nanoparticles for Optical and Electronic Applications. ACS Appl. Nano Mater..

[cit9] Liu J. (2021). *et al.*, Enhanced Vis-NIR light absorption and thickness effect of Mo-modified SnO_2_ thin films: a first principle calculation study. Results Phys..

[cit10] Dua L., Biswas P. K. (2013). Synthesis and characterization of nanostructured Mn(II) doped antimony-tin oxide (ATO) films on glass. Appl. Surf. Sci..

[cit11] Saravanakumar M. (2015). Effect of Mn Doping on the Structural, Optical and Magnetic Properties of SnO_2_ Nanoparticles by solvothermal processing. Acta Phys. Pol. A.

[cit12] Shao C., Chen A., Yan B., Shao Q., Zhu K. (2016). Improvement of electrochemical performance of tin dioxide electrodes through manganese and antimony co-doping. J. Electroanal. Chem..

[cit13] Ragupathy S., Ramasundaram S., Thennarasu G., Harishsenthil P., Krishnakumar M., Hwan Oh T. (2023). Effect of Mn doping on structural, optical and photocatalytic properties of SnO_2_ nanoparticles. Ceram. Int..

[cit14] Liu J. (2021). *et al.*, Highly efficient photocatalytic degradation of oil pollutants by oxygen deficient SnO_2_ quantum dots for water remediation. Chem. Eng. J..

[cit15] Rajeswaran P., Shanmuganathan M., Shanmuga sundari T., Elavarasan A., Sivakarthik P. (2023). A simple fabrication of Mn doped SnO_2_ nano particles towards improved Congo red degradation photocatalytic activity. Mater. Today: Proc..

[cit16] Shao J. (2021). *et al.*, Aqueous synthesis of Nb-modified SnO_2_ quantum dots for efficient photocatalytic degradation of polyethylene for *in situ* agricultural waste treatment. Green Process. Synth..

[cit17] Negi S., Sharma A., Sharma P. (2020). Investigation of Magnetic and Optical Properties of Mn-Doped SnO_2_ at Different Annealing Temperatures. J. Sci. Res..

[cit18] Ahmad T., Ansari M. Z. (2022). Enhancement of infrared shielding property of SnO_2_ using Sb as a dopant. Mater. Res. Express.

[cit19] Gawade V. V., Sabale S. R., Dhabbe R. S., Garadkar K. M. (2023). Environmentally sustainable synthesis of SnO_2_ nanostructures for efficient photodegradation of industrial dyes. J. Mater. Sci.:Mater. Electron..

[cit20] Usha K. S., Prasath G. V., Yeol S. (2024). Structural and Magnetic Behavior of Mn-Doped SnO_2_ Nanorods for Diluted Magnetic Semiconductors. Appl. Phys. A.

[cit21] Leonardy A., Hung W., Tsai D., Chou C. (2009). Structural Features of SnO_2_ Nanowires and Raman Spectroscopy Analysis. Cryst. Growth Des..

[cit22] Wang X., Chen A., Wu X., Zhang J., Dong J. (2024). Synthesis and Modulation of Low - Dimensional Transition Metal Chalcogenide Materials *via* Atomic Substitution. Nano-Micro Lett..

[cit23] Usha K. S., Prasath G. V., Lee S. Y., Lee S. Y., Nadu T. (2024). Appl. Phys. A.

[cit24] Vallimeena S., Helina B. (2023). Cauliflower-like strontium-doped SnO_2_ nanoparticles for photocatalytic degradation. Mater. Sci. Technol..

[cit25] Surendhiran K. C. S. S., Kumar P. M., Kumar E. R., Khadar Y. A. S. (2020). Green synthesis of – SnO_2_ nanoparticles using *Delonix elata* leaf extract: evaluation of its structural, optical, morphological and photocatalytic properties. SN Appl. Sci..

[cit26] Fouad Z. K. H., Mohamed H. K. S., Ansari H. Z. A. (2020). Feasibility study of doped – SnO_2_ nanomaterial for electronic nose towards sensing biomarkers of lung cancer. J. Mater. Sci.:Mater. Electron..

[cit27] Akkera H. S., Mann V., Varalakshmi B. N., Ploloju M., Kambhala N., Venkatesh G. (2023). Effect of Sr-doped on physical and photoluminescence properties of SnO_2_ transparent conducting oxide thin films. J. Mater. Sci.:Mater. Electron..

[cit28] Ragupathy S., Ramasundaram S., Thennarasu G., Harishsenthil P., Krishnakumar M., Hwan T. (2023). Effect of Mn doping on structural, optical and photocatalytic properties of SnO_2_ nanoparticles. Ceram. Int..

[cit29] Arif M. (2022). *et al.*, High photocatalytic performance of copper-doped SnO_2_ nanoparticles in degradation of Rhodamine B dye. Opt. Mater..

[cit30] Online V. A. (2014). Influence of manganese ions in the band gap of tin oxide nanoparticles: structure, microstructure and optical studies. RSC Adv..

[cit31] Li X., Qian J., Li J., Xu J., Xing J., Liu L. (2019). A facile synthesis of antimony – doped tin oxide – coated – TiO_2_ composites and their electrical properties. J. Mater. Sci.:Mater. Electron..

[cit32] Costa I. M., Colmenares Y. N., Pizani P. S., Leite E. R., Chiquito A. J. (2018). Sb doping of VLS synthesized SnO_2_ nanowires probed by Raman and XPS spectroscopy. Chem. Phys. Lett..

[cit33] Gupta P., Rathore V., Sahoo S., Majumder S. (2023). Investigation of electronic properties of Mn doped SnO_2_ thin film. Vacuum.

[cit34] Nogueira D. S. C., Franceschini D. F., Ponzio E. A. (2020). Tuning the morphology of manganese oxide nanostructures for obtaining both high gravimetric and volumetric capacitance. Mater. Adv..

[cit35] Wei D. G. (2026). *et al.*, Dual oxygen-mediated charge transfer and ultrafast transient behaviors in Ti_3_C_2_(−O)@MOS heterostructures: enhancing photoelectrochemical performance. Appl. Surf. Sci..

[cit36] Wu D. (2025). *et al.*, Weak sunlight responsive photocatalytic degradation *via* synergy of oxygen vacancies and S-scheme heterojunctions in g-C_3_N_4_-Zn_2_SnO_4_. J. Photochem. Photobiol., A.

[cit37] Kumar A. (2025). *et al.*, Defect engineering approaches for metal oxide semiconductor-based chemiresistive gas sensing. Coord. Chem. Rev..

[cit38] Liu J., Zhai Z., Jin G., Wu L., Gao F., Hong W. (2020). Numerical Analysis of Oxygen Vacancy Distribution in Semiconductor Gas Sensors in the Cooling Process Based on the Model of Gradient-Distributed Oxygen Vacancies. Mater. Sci..

[cit39] Gaber A., Abdel-Rahim M. A., Abdel-Latief A. Y., Abdel-Salam M. N. (2014). Influence of Calcination Temperature on the Structure and Porosity of Nanocrystalline SnO_2_ Synthesized by a Conventional Precipitation method. Int. J. Electrochem. Sci..

[cit40] Tan W., Ruan X., Yu Q., Yu Z., Huang X. (2015). Fabrication of a SnO_2_-based acetone gas sensor enhanced by molecular imprinting. Sensors.

[cit41] Liu J. (2020). *et al.*, Size effect on oxygen vacancy formation and gaseous adsorption in ZnO nanocrystallites for gas sensors: a first principle calculation study. Appl. Phys. A.

[cit42] Chiu H., Yeh C. (2010). Hydrothermal Synthesis of SnO_2_ Nanoparticles and Their Gas-Sensing of Alcohol. J. Phys. Chem. C.

[cit43] Khan S., Noor T., Iqbal N., Yaqoob L. (2024). Photocatalytic Dye Degradation from Textile Wastewater: A Review. ACS Omega.

[cit44] Renuga R., Srinivasan S., Thangeeswari T., Suresh S., Bomila R. (2022). Effect of dopant concentration Mn in SnO_2_ nanoparticles on photocatalytic, magnetic and optical properties. Digest Journal of Nanomaterials & Biostructures.

